# Associations of Monitor-Assessed Activity with Performance-Based Physical Function

**DOI:** 10.1371/journal.pone.0153398

**Published:** 2016-04-13

**Authors:** Natasha Reid, Robin M. Daly, Elisabeth A. H. Winkler, Paul A. Gardiner, Elizabeth G. Eakin, Neville Owen, David W. Dunstan, Genevieve N. Healy

**Affiliations:** 1 School of Public Health, The University of Queensland, Brisbane, Australia; 2 Centre for Physical Activity and Nutrition Research, Deakin University, Melbourne, Australia; 3 Mater Research Institute, The University of Queensland, South Brisbane, Australia; 4 Baker IDI Heart and Diabetes Institute, Melbourne, Australia; 5 Melbourne School of Population and Global Health, The University of Melbourne, Melbourne, Australia; 6 Department of Medicine, Alfred Hospital, Monash University, Melbourne, Australia; 7 School of Public Health & Preventive Medicine, Monash University, Melbourne, Australia; 8 School of Sport Science, Exercise and Health, The University of Western Australia, Perth Australia; 9 Mary MacKillop Institute of Health Research, Australian Catholic University, Melbourne, Australia; 10 School of Physiotherapy, Curtin University, Perth, Australia; 11 Centre for Research in Geriatric Medicine, The University of Queensland School of Medicine, Brisbane, Australia; University of Tuebingen, GERMANY

## Abstract

The purpose of this study was to investigate the cross-sectional associations of monitor-derived measures of sedentary time and physical activity with performance-based physical function in healthy Australian adults. Data from 602 participants (mean age 58.1±10.0 years; 58% female) from the 2011/12 wave of the Australian Diabetes, Obesity and Lifestyle (AusDiab3) study were analyzed. The thigh-worn activPAL3^™^ monitor (7-days continuous wear) was used to derive time during waking hours spent: sitting/reclining; standing; and, stepping (overall, and separately as light [<3 METs] and moderate-to-vigorous physical activity [MVPA; ≥3 METs]), and number of sit-stand transitions. Associations of these (in hours/day, or 15 transitions/day) with physical function measures (8ft Timed Up and Go [TUG-8; log-transformed seconds] and Knee Extensor Strength [KES; kg]) were tested via linear regression, adjusting for confounders. Interactions by sex and age-category (<45; 45–54; 55–64; ≥65 years) were tested. In all participants, KES was significantly (p<0.05) associated with stepping and MVPA stepping only; none of the activity measures were associated with TUG-8. However, subgroup analysis revealed that in older adults (≥65 years), TUG-8 was associated with stepping and MVPA stepping (both p<0.05). All associations with sitting time, standing, sit-stand transition and sex interactions were not statistically significant. In summary, sitting time was not significantly associated with impaired muscle strength or gait/mobility in Australian adults aged 36–80 years, but light- to moderate activity (stepping) was positively associated with muscle strength, and gait/mobility in older adults aged ≥65 years. The direction of causation is not known and remains important to investigate considering the high prevalence of both poor function and limited activity in older age.

## Introduction

Deterioration in physical function, which represents a reduced capacity to undertake activities of everyday living, occurs in approximately 20% of healthy adults aged 60 years and older, and increases to approximately half of all adults aged 80 years and older [1]. From a clinical perspective, this deterioration in physical function is important because it has been associated with a range of adverse health outcomes, including an increased risk of falls and resultant fractures [2], which can lead to a loss of independence and reduced quality of life [3]. Specifically, reduced lower-extremity muscle strength and gait speed have been consistently associated with an increased risk of falls [4], reduced mobility [5] and premature mortality [6]. Thus, understanding and addressing behaviours that can improve or maintain lower extremity muscle strength and function across the life span is important in order to reduce disease and disability risk, enabling older adults to live independently longer [3].

Physical activity is widely recommended to maintain overall health and physical function in older adults. However, most research to date has focused on the relationship between physical function and activity at the higher end of the intensity spectrum—moderate- to vigorous-intensity physical activity (MVPA). Despite the well-established health benefits of MVPA [7], this only constitutes a very small proportion of waking hours—on average less than 5% in the general adult population, and less than 2% in adults aged 65 years and older [8]. Rather, the majority of time for many older adults is spent in behaviours that fall within the sedentary or light intensity range, such as sitting, standing or light ambulatory activities [9]. As such, it is important to evaluate whether these behaviours are associated with physical function, either beneficially or detrimentally.

A number of studies have examined the associations between time spent in sedentary or light intensity activities with physical function. A study in 862 adults aged 65 years and older observed that accelerometer-derived time spent in activities at the upper-end of the light intensity range (i.e., 1,041–1,951 counts/minute) was significantly and positively associated with improved physical health, including self-reported lower-extremity function [10]. Whether there is a relationship between sedentary time (sitting or reclining with low energy expenditure while awake) [11] and muscle strength or functional performance is less clear. From a physiological perspective, there is sound rationale to suspect that reduced muscle contractile activity during sitting [12] especially for prolonged periods, may lead to skeletal muscle atrophy and ultimately reduced muscle strength and/or impaired function. Whether habitual upright activity (standing and walking) can prevent muscle atrophy or deterioration in muscle function is uncertain, as previous cross-sectional studies have reported mixed results, with some observing detrimental associations [13, 14] while others report no significant associations [15] and even positive associations [16]. The reason for these apparent discrepancies may relate to differences in participant characteristics between studies (e.g., older vs. middle aged, functional status), limitations related to the research design (e.g., possibility of reverse causation), and/or the measures used to quantify the sedentary time (e.g. television viewing time, total sitting time, objective measures of sedentary time).

To date, the majority of studies examining the association of activity with physical function have used self-report data of exposures, outcomes or both, or objective measures from monitors that cannot directly measure sedentary versus upright posture (e.g., hip- or wrist- worn accelerometers). Few studies have used monitors that can distinguish posture accurately, such as the thigh-worn activPAL3^™^ monitor, and those that have utilised both objective measures of activity and objective measures of function have primarily been conducted in old (or very old) people [13, 14, 17, 18]. The relationship of activity (across the intensity spectrum) with physical function across the life span is less clear. Examining this relationship across ages will provide greater insight into variation in behaviour within the normal range of functioning.

To address these evidence gaps, the aim of this study was to investigate the associations of *posture-based*, *objective* measures of time spent sitting, sitting for prolonged periods (≥30 minutes at a time), standing, stepping (overall, and at a light [<3 MET] and at least moderate [≥3 MET] cadence), as well as sit-stand postural transitions with *performance-based* lower-extremity muscle strength and gait speed/mobility in a sample of healthy community dwelling adults (36–80 years). Given the established sex and age-related differences in activity [8] and functional capacity [19], we also tested for potential sex and age group interactions of associations of activities with physical function. It was hypothesized that all activities would be associated with function measures, with sitting (and prolonged sitting) expected to be adversely associated, while standing, stepping and sit-stand transitions would be beneficially associated.

## Materials and Methods

### Participants

The Australian Diabetes, Obesity and Lifestyle Study (AusDiab) is a longitudinal study examining the history of diabetes, pre-diabetes, heart disease and kidney disease in community dwelling Australian adults. As previously reported [20], baseline data was collected in 1999–2000 from those aged at least 25 years using a probabilistic sampling frame (n = 11,247). Since then, two additional waves of data collection have occurred (2004/05 and 2011/12) and for this study we have used data collected from the 2011/12 wave. The study was approved by the Alfred Health Human ethics committee, and signed informed consent was obtained by all participants.

### Data collection

At each wave, participants attended a testing site where biochemical, anthropometric, and behavioural assessments were conducted, and questionnaire data were collected. In 2011/12, 4,614 participants across 46 sites in Australia attended the on-site testing [20]. The representativeness of this third wave of attendees has previously been reported, with on-site attendees being on average younger, with lower BMI, better cardiovascular health, higher education and lower rates of smoking, compared to non-attendees [20].

At this third wave of data collection, physical function assessments (for all participants who were able to complete the tests) and activity monitor assessments (on a sub-sample) were added to the on-site testing protocol. On each testing day, up to five participants were recruited for the activity monitor sub-study, beginning with the first potentially eligible participant (i.e., ambulatory, not pregnant). Participants were informed that the activity monitors would collect data on sitting, standing, and stepping time over seven consecutive days. Participation in this component required additional informed written consent. Of the 1014 participants approached, 784 agreed to participate, and 741 provided at least one valid day of activity monitor data. Of these, those with missing values for outcome variables (n = 84) and covariates (n = 55) were excluded, leaving data from 602 adults available for full analysis (final sample).

### Activity outcomes

The activPAL3^™^ monitor is a small (53 × 35 × 7 mm; 15 g), unobtrusive device that uses raw data collected (at 20 Hz) on thigh angle and acceleration to identify periods spent sitting/reclining (herein referred to as ‘sitting’), standing, and stepping, stepping speed (cadence), and step counts, using proprietary algorithms. Sitting and lying down may not relate equally to physical function, and a recent small-scale validation study showed the activPAL3^™^ can distinguish between these postures to some degree [21]. We chose to examine original activPAL3^™^ classifications as these have been more extensively validated than sitting and lying as separate activity classifications. This monitor has been shown to be highly accurate (overall observer-monitor activity agreement of 95.9% for the assessment of second-by-second of sitting, standing and stepping activity; [22, 23]) and reliable (interclass correlation of 0.79 to 0.99 for inter-observer reliability) [24] in both adults and older adults [25]. The monitor was initialised (default settings) then fitted to the anterior midline of the participant’s right thigh. Participants were instructed to wear it continuously (24 hours/day, without removing for showering/bathing) for seven consecutive days, complete a sleep and wear diary, and post the monitor and diary back to AusDiab3 research staff in a reply-paid envelope.

The data were downloaded using the proprietary software (version 6.4.1) and processed using a customised program in SAS 9.3 (SAS Institute Inc., Cary, NC, USA) that combined activPAL3^™^ and diary data. If not reported, apparent sleep/wake times were estimated based on visual scanning of the data for cessation/resumption of standing or stepping preceding/following prolonged periods of sitting. All data were visually inspected and corrected as required for any unreported long removal periods. Consistent with the events-based approach [26], and to correct for imprecisions in diary reporting, whole bouts of activity (rather than times) were classed as awake/not and removed/not, with bouts that were mostly (≥50%) awake/removed being initially classed as such. To remove any waking activity within these sleeping periods from imprecise reporting, any bout of activity <20 minutes at the beginning and end of each initially identified sleep period was reclassified as awake. Wear days were then identified (from wake time until wake time the following day) and these were considered valid if the monitor was worn for ≥10 h (when waking hours were not reported in the diary) and for ≥80% of waking hours. For each valid day, the sum total of waking wear time, time spent sitting, sitting in prolonged bouts, standing, stepping and number of sit-stand transitions was calculated. Due to the possibility that only particular stepping speeds are beneficial to function (i.e., those consistent with moderate activity), we also investigated slow and fast stepping separately, split at 3 METs (as estimated by the device from cadence) to distinguish at least moderate stepping (MVPA stepping) from light stepping. These were averaged across valid days, and standardised for waking wear time by the residuals method [27].

### Physical function

Physical function was measured objectively using the 8ft Timed Up and Go (TUG-8) test [28] and the Knee Extensor Strength (KES) test [29]. A shorter time to complete the TUG-8 test (in seconds), which was measured by stopwatch, indicates better dynamic gait speed and mobility across a combination of three commonly performed functional activities of daily living (sitting, standing, walking and turning). Participants started by sitting in a chair that was placed at the end of a marked 8ft/2.44-meter walk. On the command ‘Go’, participants were asked to rise from the chair, walk at a comfortable speed for 2.44 meters, turn around, and walk back and sit down in the chair. This test has shown good reliability (ICC = 0.95), and relative validity against gait speed as a criterion (*r* = 0.61) [28].

The KES is a measure of lower-limb isometric muscle strength, with greater force (in kg) indicating better knee extensor strength. KES was measured using Lord's strap assembly incorporating a strain gauge (Neuroscience Research Australia, Sydney, Australia). Briefly, participants were seated on a stool with their hip and knee at a 90 degree angle, and a webbing strap with a Velcro fastener attached to their dominant leg approximately 5–10 cm above the ankle joint. After one practice trial and a one-minute rest, participants performed their two test trials by extending their leg against the strap with maximal force for 2–3 seconds, having been instructed to contract as fast and as forcefully as possible [29]. Thigh length (i.e., from hip to knee) was also measured. This test has been shown to have good test-retest reliability (ICC > 0.9) [30] and good construct validity with other measures of muscle strength (*r* = 0.768) [29]. The KES test is reported in total kilograms, adjusted for thigh length.

Self-reported physical function (PF-10; unstandardized) was obtained from the 10 physical function specific items in the SF-36 quality of life questionnaire and used as a descriptive measure [31].

### Variables considered as confounders

Interviewer-administered questionnaires were used to obtain socio-demographic information on age, sex, marital status, housing status, income, smoking status, and country of birth (collapsed later into Australia or New Zealand versus other). Information was also collected on work status, family history of diabetes, and self-rated health (see Table 1 for response categories). Alcohol and energy intake (g/day) were determined using a self-administered validated food frequency questionnaire (average during the past 12 months) [32]. Body Mass Index (BMI; kg/m^2^) was obtained by recording height (nearest 0.5cm) and weight (nearest 0.5kg) via a stadiometer and scales respectively, using standardized protocols [33]. Depressive symptoms were evaluated using the Center for Epidemiologic Studies Depression Scale (CESD; 0–20) [34]. Scores were broken down into three categories: no depressive symptoms (<10); mild depressive symptoms (10–14); and severe depressive symptoms (>14) [35].

**Table 1:** Characteristics of AusDiab 2011/12 included participants (n = 602 in the monitor subsample with relevant data) and remaining testing site attendees (n = 4012 Australian adults).

Variables	Included participants (n = 602)	Remaining AusDiab3 on-site attendees (n = 4012)	*p* (included vs excluded)
*Characteristics*			
Age (years); mean (SD)	58.1 (10.0)	61.2 (11.4)	<0.001
35 to 45; years; n (%)	60 (10.0)	293 (6.4)	
45 to <55; n (%)	174 (28.9)	874 (18.9)	
55 to <65; n (%)	203 (33.7)	1339 (29.0)	
≥65; n (%)	165 (27.4)	1506 (32.6)	
Female; n (%)	352 (58.47)	2200 (54.8)	0.076
Owns dwelling; n (%)	537 (89.2)	3506 (87.4)	0.153
Australian/NZ; n (%)	492 (81.7)	3126 (67.8)	0.025
Yearly household income			0.930
Low, <$30k	86 (14.3)	732 (18.2)	
Mod-low, $30 to <$60k	147 (24.4)	886 (22.1)	
Mod-high, $60 to <100k	127 (21.1)	785 (19.6)	
High, ≥ $100k	208 (34.6)	1132 (28.2)	
Employment Status			0.012
Full Time	226 (37.5)	1230 (30.7)	
Part Time	136 (22.6)	820 (20.4)	
Retired	176 (29.2)	1366 (34.1)	
Other	64 (10.6)	417 (10.4)	
BMI category; n (%)			0.526
Underweight/Normal; <25	195 (32.4)	1254 (31.3)	
Overweight; 25 to < 30	258 (42.9)	1641 (40.9)	
Obese; ≥30	149 (24.8)	1107 (27.6)	
Self-rated health; n (%)			0.007
Excellent	77 (12.8)	418 (10.4)	
Very good	250 (41.5)	1501 (37.4)	
Good	227 (37.7)	1561 (38.9)	
Fair/poor	48 (8.0)	488 (12.2)	
Alcohol Intake; n (%) ^a^			0.563
None/Low	70 (11.6)	490 (12.2)	
Normal	391 (65.0)	2137 (53.3)	
High	75 (12.5)	556 (13.9)	
Severe	66 (11.0)	411 (10.2)	
Family history of diabetes; n (%)	172 (28.6)	1130 (28.2)	0.830
Center for Epidemiologic Studies Depression Scale (0–20); n (%)			0.123
No symptoms (<10)	551 (91.5)	3444 (74.6)	
Mild symptoms (10 to14)	32 (5.3)	279 (6.0)	
Severe symptoms (>14)	19 (3.2)	154 (3.3)	
*Physical function* measures; median (25^th^ to 75^th^ Percentile)			
Timed up and Go (s)	5.6 (4.9 to 6.5)	5.9 (5.1 to 7.0)	<0.001
Knee extensor strength test (kg)	24.2 (16.6 to 34)	23.3 (15.5 to 33.2)	0.424
Physical function (PF-10) ^b^	90 (80 to 100)	90 (70 to 95)	<0.001
*Activity variables* ^c^			
Sitting, all, hrs/day	8.7 (1.8)	-	-
Prolonged Sitting, h/day ^d^	4.0 (1.7)	-	-
Standing, h/day	4.9 (1.5)	-	-
Stepping, all, h/day	2.0 (0.6)	-	-
Light stepping, h/day ^e^	1.0 (0.4)	-	-
MVPA stepping, h/day ^f^	1.0 (0.4)	-	-
Sit-stand transitions, mean (SD) ^g^	53.3 (14.8)	-	-

^a^ low = 0 g/day, normal = <25 g/day (men) & < 15 g/day (women), high = 25–<45 g/day (men) & 15– < 25 g/day (women), severe = >45 g/day (men) & >25 g/day (women);

^b^ self-reported physical function obtained from the 10 physical function specific items in the SF-36 quality of life questionnaire;

^c^ all objective activity variables standardised for worn waking time;

^d^ >30 minutes uninterrupted sitting;

^e^ Light stepping is <3 METs;

^f^ MVPA (moderate-to-vigorous physical activity) stepping is at ≥ 3 METs;

^g^ Sit-stand transitions adjusted for sitting; statistically significant differences between the final sample and the remaining on-site attendees were evaluated using logistic regression analyses (survey commands).

### Sample size analysis

*A priori* calculations in G*Power version 3.2.7 (Heinrich Heine University, Dusseldorf, Germany) showed that approximately 530 participants would be required to provide 90% power to detect small effect sizes (Cohen’s *f*^2^ = 0.02) [36], with 5% significance 2-tailed; this was consistent with the approximate size available from the AusDiab3 monitor subsample.

### Statistical analyses

Data processing and analyses were performed in STATA (version 12, College Station, TX, Stata Corporation), using survey commands with linearized variance estimates in view of the clustered, stratified design. Statistical significance was set as two-sided *p*<0.05 (including for interaction terms), with *p*<0.1 used as a reporting threshold for interactions. Descriptive statistics are presented as means and standard deviations (SD) for normally distributed data, median (25^th^, 75^th^ percentile) for non-normal continuous data, or percentages for categories. Characteristics of included participants (n = 602 from the subsample, with complete data) and remaining AusDiab3 onsite attendees (n = 4012 from all who attended a tested site, not including those in the final sample) are described and the association of these characteristics with study inclusion were tested using logistic regression (survey commands).

Multivariable linear regression analyses examined the associations of time spent per day in the three main activities (sitting, standing, stepping), sub-types of these activities (prolonged sitting, light stepping, MVPA stepping) and postural transitions with time to complete the TUG-8 test (in seconds) and weight lifted on the KES test (in kg), adjusting for potential confounders. Models examining postural transitions also adjusted for sitting time. To conform to modelling assumptions (normality and heteroscedasticity), TUG-8 time was log transformed. Models did not display non-normality or heteroscedasticity. Non-linearity in associations of activity with the outcomes was not evident in scatterplots of residuals versus the activities. To account for the many participants who reached the KES test limit (60 kg), truncated regression models were also used (see S1 Table), as a sensitivity analysis only as these models did not correct for clustering and stratification. All models adjusted, regardless of significance, for age, sex, BMI and self-rated health and, in KES models, for thigh length (to account for differences in lever-arm length). Variables associated with the outcome at p<0.2 in backwards elimination models were also included (namely, depressive symptoms and alcohol intake in TUG-8 models and employment status in KES models). Interactions by age group (36–44; 45–54; 55–64; ≥65 years) and sex were also tested using regression models.

## Results

Table 1 presents the characteristics and comparison of the AusDiab3 on-site attendees who were and were not included in the current analyses. The final sample ranged in age from 36 to 80 years, 58% were women, 34% had a high (≥$100K) household income, 65% with normal (<25 g/day for men and <15 g/day for women) alcohol intake, 41% with very good self-rated health, 34% were categorised as overweight, and 91% were without depressive symptoms. Included participants were significantly younger (p<0.001), had higher income (p<0.001), full-time employment (p = 0.011), higher self-rated health (p = 0.005), and shorter time to complete the TUG-8 test (p<0.001) compared to participants not included. 496 participants provided 7 days of monitor data (65 provided 6 days, 15 provided 5, 10 provided 4, 9 provided 3, 4 provided 2, and 3 participants provided 1 day of data). Findings were not meaningfully different for those with 7 days compared to those with less data; hence, to maximize sample size, all those with at least 1 day of data were included in the study. Participants wore the monitor for an average (mean ± SD) of 15.7±1.1 h/day during waking hours, of which an average 55.4% (8.7±1.8 h/day) was spent sitting, (including 25.5% in prolonged sitting [4.0±1.7 h/day]), 31.2% (4.9±1.5 h/day) was spent standing, and 12.7% (2.0±0.6 h/day) was spent stepping (with 6.6% spent in both light and MVPA stepping). Participants performed an average of 53±14 sit-stand transitions per day. Characteristics and comparisons between age categories are available in S2 Table.

### Association of activity measures with physical function

In the overall sample, no statistically significant associations were found between any type of activity with TUG-8, nor of sitting, prolonged sitting or standing with KES (Table 2). Significantly higher KES (β [95%CI] kg) was observed with each additional hour per day of stepping (2.3 [0.8, 3.7] kg, p = 0.003), light stepping (2.9 [0.3, 5.5] kg, p = 0.030) and of MVPA stepping (3.7 [0.9, 6.4] kg, *p* = 0.009). Correcting for the upper test limit in the sensitivity analyses (see S1 Table) mostly did not affect conclusions except that the association of light stepping with KES was slightly weaker and no longer statistically significant (2.25 [-0.10, 4.61] kg, *p* = 0.061).

**Table 2:** Association of activPAL3^™^ derived activities with the 8ft Timed Up and Go (TUG-8) and Knee Extensor Strength (KES) test in Australian adults aged 36–80 years.

	TUG-8 completion time (seconds) RR (95% CI) ^a^	*p*-value	KES (kilograms) β (95% CI) ^b^	*p*-value
Sitting (all), h/day	1.01 (1.00 to 1.02)	0.245	-0.30 (-0.70 to 0.09)	0.131
Prolonged Sitting, h/day ^c^	1.00 (0.99 to 1.02)	0.474	-0.40 (-0.93 to 0.13)	0.134
Standing, h/day	0.99 (0.98 to 1.01)	0.329	0.01 (-0.43 to 0.44)	0.980
Stepping (all), h/day	0.98 (0.95 to 1.02)	0.341	2.28 (0.82 to 3.74)	0.003
Light stepping, h/day ^d^	0.98 (0.93 to 1.03)	0.378	2.90 (0.28 to 5.51)	0.030
MVPA stepping, h/day ^d^	0.97 (0.92 to 1.03)	0.383	3.68 (0.96 to 6.40)	0.009
Sit-stand transitions, 15 transitions/day	1.00 (1.00 to 1.00)	0.961	0.04 (-0.01 to 0.09)	0.097

^a^ Back-transformed from log-transformed outcome as Relative Rate (RR) with 95% confidence interval (CI) obtained in linear regression analyses (STATA ‘survey commands’) that corrects for clustering/stratification and adjust for age (years), sex (male/female), self-rated health (excellent, very good, good, fair/poor), depressive symptoms (none, mild, severe) and alcohol intake (none/low, normal, high, severe);

^b^ Regression coefficient (β) with 95% confidence interval (CI) that adjusts for age (years), sex (male/female), self-rated health (excellent, very good, good, fair/poor), employment status (full time, part time, retired, other) and thigh length (cm) and correct for clustering/stratification (linear regression, STATA ‘survey commands’);

^c^ Prolonged sitting = sitting uninterrupted in ≥30 minute bouts at a time;

^d^ Light stepping is <3 METs; MVPA stepping is at ≥ 3 METs.

### Age and sex variations in associations of activity measures with physical function

**Fig 1:**
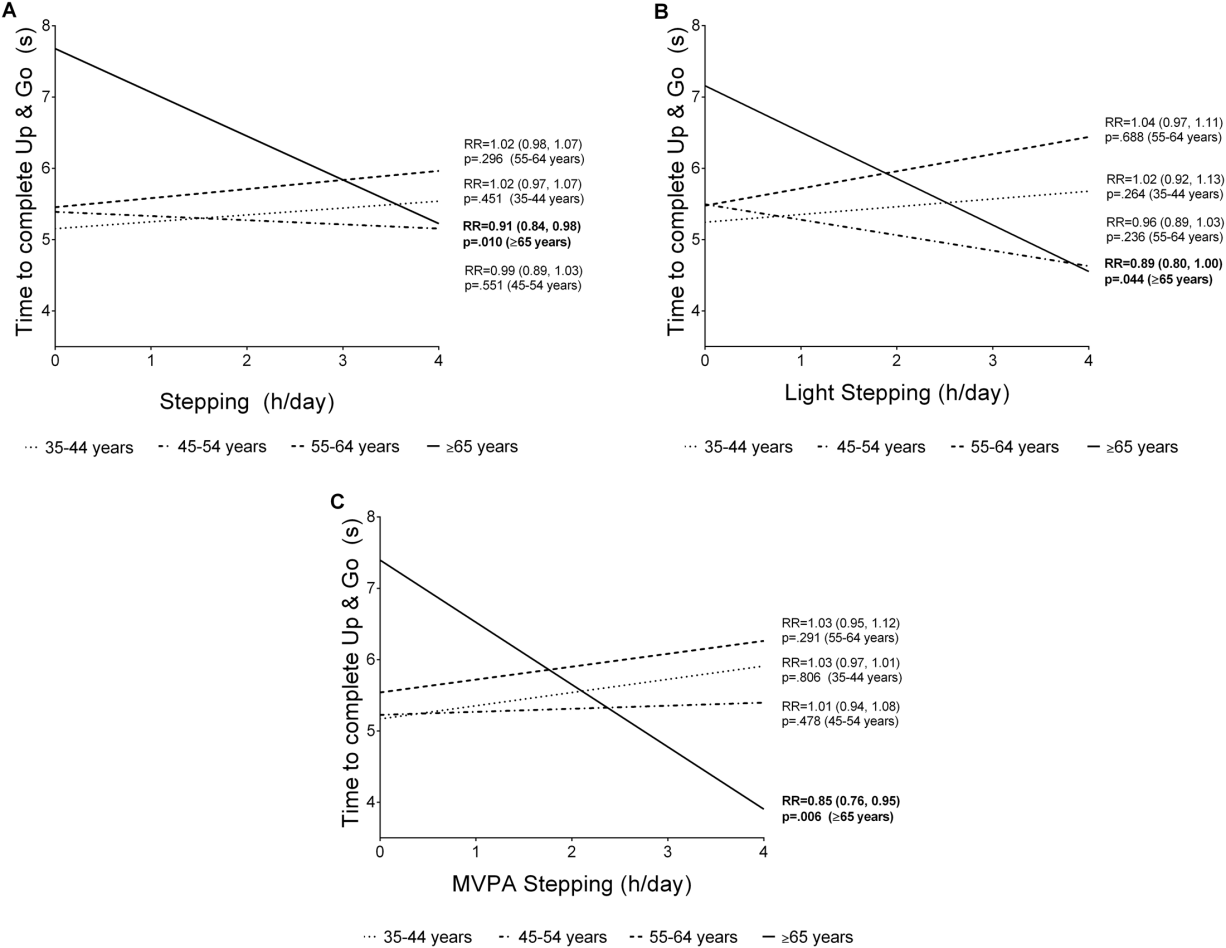
Age-group interactions for the association of Timed Up and Go (TUG-8) performance and overall stepping (A), light stepping (B), and MVPA stepping (C) in Australian adults aged 36–80 years.

Associations of activity with TUG-8 and KES did not vary significantly by sex (all p>0.05, see S3 Table). Associations of activity with performance on the KES test did not differ significantly by age (see S4 Table). There was some evidence of variation by age in the associations of TUG-8 with stepping (*p* = 0.039), light stepping (*p* = 0.052), and MVPA stepping (*p* = 0.021) only (see S4 Table, and Fig 1). For all stepping by age interactions, the effect sizes were statistically significant and strongest in those aged ≥65 years, with increased stepping time being associated with greater TUG-8 performance in this age group. Time to complete the TUG-8 test decreased significantly with each additional hour per day of stepping (by 10%, RR = 0.91, 95% CI: 0.84, 0.98, *p* = 0.010), light stepping (by 12%, RR = 0.89, 95% CI: 0.80, 1.00, *p* = 0.044) and MVPA stepping (by 18%, RR = 0.85, 95% CI: 0.76, 0.95, *p* = 0.006). See Fig 1 for further details.

## Discussion

This cross-sectional study of community-dwelling Australian adults aged 36–80 years aimed to examine the associations of posture-based, objective measures of various physical activities (from sitting to MVPA stepping) with performance-based measures of physical function. We observed that average daily stepping time was significantly associated with greater knee extensor strength (KES) in all adults aged 36–80 years. For gait and mobility (TUG-8 performance), no significant association was observed overall with stepping time, but the association varied significantly by age. Within adults aged ≥65 years only, stepping was significantly associated with a lower mean time to complete the TUG-8 test. No statistically significant associations were observed between measures of sitting time, prolonged sitting time, standing time or sit-stand transitions with TUG-8 or KES scores.

Studies using both objective and self-reported measures of activity and function have consistently shown that more activity (particularly moderate intensity) or regular structured exercise (resistance and balance training) is associated with the improvement and/or delayed onset of physical limitations [3, 13, 17, 18]. Adding to this literature, we observed a significant and positive association of both light intensity stepping (<3 METs) and MVPA stepping (≥3 METs) with faster TUG-8 time (only within adults aged 65–80 years), and between MVPA stepping and KES across all age groups (36–80 years). Our study adds to this literature by supporting the current evidence using a novel posture-based monitor not previously investigated in this setting (i.e., the activPAL3^™^). However, due to the high functional ability of the sample, the onset of physical impairment may be different to other populations. Collectively, the literature supports the promotion of activity (both light- and moderate-intensity) for the improvement of function in older adults, although future experimental or longitudinal studies would benefit from the use of high quality objective measures of activity and function to establish causal relationships.

Although it was expected that more sitting would be adversely associated with physical function, such that sitting time would increase mean TUG-8 time and decrease mean KES, no statistically significant associations were observed in this study. This could be attributed to the fact that we investigated these associations across the adult life course (e.g., in adults aged 36–80 years), and not specifically in older adults and the elderly. Further, our sample was shown to be high functioning when compared to normative scores for community dwelling older men and women [37]. Indeed, two previous studies in elderly institutionalized women aged 71–96 [13] and older adults aged 65–103 years [14] that examined this relationship using objective measures of both sedentary time and function both reported significant associations with TUG-8 [13, 14] and lower-extremity muscle strength [13]. These studies found that more sitting was associated with slower TUG-8 time and lower muscle strength. Although no such statistically significant associations were observed in our sample of healthy adults and older adults, given the potential cardiovascular and metabolic benefits of reducing sitting time [38], the message to reduce prolonged and excessive sitting time remains relevant (e.g., Australian physical activity guidelines [39]). As standing, light stepping and MVPA stepping did not show equally strong associations with physical function, people who reduce their sitting time might experience a different degree of benefit to their physical functioning depending on what they do instead of sitting. This merits further investigation.

To our knowledge, despite standing being a common activity (31% of the waking day in our sample), there has only been one other study that has investigated standing time in relation to physical function [13]. Although likely underpowered, this study of 19 institutionalized elderly women also observed no significant associations of standing with TUG-8 performance or muscle strength, as well as with balance, flexibility, maximal walking, or the chair stands test [13]. This suggests that more dynamic muscle contractions or movements may be needed to improve functional performance. However, when we examined the association between the number of daily sit-stand transitions (adjusted for sitting time) with muscle strength or gait/mobility we again observed no significant association overall or within any of the examined age groups. While similar findings were observed in study of 162 healthy community-dwelling adults aged 60 to 86 years [40], several other studies have reported that more frequent breaks in sedentary time are associated with better physical function [41]. It is evident from these findings that further research on the role of sit-stand transitions (and activity accumulation patterns more broadly), as well as standing time during free-living behaviour, is needed to investigate these elements for functional and other health benefits, particularly in adults across the age range and in multiple settings (i.e. community, assisted living, institutional, residential care).

Key strengths of this study include the objective measurement of *both* physical function and activity specifically using a novel monitor that is valid and reliable in measuring varying postures and stepping [24, 25]. Though the monitor provides accurate measures, measurement error could still have affected findings. The data reduction methods (e.g., removing sleep and non-wear) were typical of usual practice in the field [42] but have not been validated. Compliance was excellent, however, it is not completely clear whether the monitoring period is sufficient to estimate usual activity [42], possibly leading to attenuated associations of activity with physical function. MVPA and light stepping reflect faster and slower stepping, but not necessarily stepping at a true moderate to vigorous or light MET level, as the activPAL accurately measures cadence [43], but not necessarily METs. The recruitment of a geographically diverse sample of community dwelling adults across Australia was in some regards a methodological strength. However, in combination with biases in sub-sample recruitment, participation, and eligibility to wear the monitor, this meant that few participants were at the older end of the age spectrum (i.e., ≥65), with none >80 years. The oldest old are those in whom associations have been observed previously and for whom associations might be strongest, given the greater degree of physical limitations that occur with ageing. Findings from this study are not generalizable beyond the ages covered. The cross-sectional nature of the study means the direction of the relationship of physical activity with physical function could not be inferred, with bi-directional relationships and reverse causation both distinct possibilities. Further, while the study was estimated to have enough participants to detect associations of a “small” effect size (f^2^ = 0.02), this is the overall effect size that did not account for clustering, may not be sufficient for smaller group sizes, and is not to say that associations smaller than this amount (for which the study was not adequately powered) have no clinical relevance. Clinically meaningful differences on the TUG-8 and KES tests are not established.

In summary, we found higher levels of ambulatory activity (particularly moderate-intensity activity) tended to be associated with better physical function, particularly in adults aged 65 years and older. We did not observe significant associations of sitting time, prolonged sitting time, standing, or number of sit-stand transitions with lower-limb muscle strength or gait/mobility in our sample of adults aged 36–80 years. Future studies should focus on composite measures of function (e.g., sarcopenia) that can be examined over a wide range of impairments. Future studies should also include all ages (particularly the oldest-old adults) and use longitudinal and/or intervention designs, when feasible, to examine evidence regarding traditional, bi-directional and reverse-causal relationships.

## Supporting Information

S1 Table Association of activPAL3^™^ derived activities with the Knee Extensor Strength (KES; truncated model analysis) test in Australian adults aged 36–80 years. (DOCX)

S2 Table Characteristics of participants included in the study (n = 602) by age group (AusDiab 2011/12). (DOCX)

S3 Table Associations of sitting, prolonged sitting, standing, stepping, light stepping, MVPA stepping and sit-stand transitions with the 8ft Timed Up and Go (TUG-8) and Knee Extensor Strength (KES) test in Australian men (n = 250) and women (n = 352) aged 36–80 years (AusDiab 2011/12). (DOCX)

S4 Table Associations of stepping, light stepping, MVPA stepping and sit-stand transitions with the 8ft Timed Up and Go (TUG-8) and Knee Extensor test (KES) within various age-groups in Australian adults aged 36–80 years. (DOCX)
