# Supplementary material for: Associations of Monitor-Assessed Activity with Performance-Based Physical Function
Source: PLoS One. 2016 Apr 13;11(4):e0153398. doi: 10.1371/journal.pone.0153398 (PMC4830578; doi:10.1371/journal.pone.0153398)
Supplement: S1 Table — (DOCX) [file pone.0153398.s001.docx]

**S1 Table. Association of activPAL3^TM^ derived activities with the Knee Extensor Strength (KES; truncated model analysis) test in Australian adults aged 36–80 years.**

|  | **Knee Extensor Strength**  **Truncated Regression (kilograms)**  **β (95% CI) ^a^** | ***p*-value** |
| --- | --- | --- |
|  |  |  |
| Sitting (all), h/day | -0.23 (-0.70 to 0.25) | 0.346 |
| Prolonged Sitting, h/day ^b^ | -0.30 (-0.79 to 0.20) | 0.239 |
| Standing, h/day | 0.03 (-0.54 to 0.60) | 0.922 |
| Stepping (all), h/day | 1.66 (0.33 to 3.00) | 0.014 |
| Light stepping, h/day ^c^ | 2.25 (-0.1 to 4.61) | 0.061 |
| MVPA stepping, h/day ^c^ | 2.62 (0.39 to 4.86) | 0.021 |
| Sit-stand transitions, 15 transitions/day | 0.22 (-0.65 to 1.09) | 0.622 |

This is the S1 Table legend.

^a^ Regression coefficient (β) with 95% confidence interval (CI) that adjusts for age (years), sex (male/female), self-rated health (excellent, very good, good, fair/poor), employment status (full time, part time, retired, other) and thigh length (cm) and corrects for the 60 kg upper limit of the test (truncated regression, sensitivity analysis); ^b^ Prolonged sitting = sitting uninterrupted in ≥30 minute bouts at a time; ^c^ Light stepping is <3 METs; MVPA (moderate-to-vigorous physical activity) stepping is at ≥ 3 METs.
