# Supplementary material for: Associations of Monitor-Assessed Activity with Performance-Based Physical Function
Source: PLoS One. 2016 Apr 13;11(4):e0153398. doi: 10.1371/journal.pone.0153398 (PMC4830578; doi:10.1371/journal.pone.0153398)
Supplement: S2 Table — (DOCX) [file pone.0153398.s002.docx]

**S2 Table. Characteristics of participants included in the study (n=602) by age group (AusDiab 2011/12).**

| **Characteristic** | **35–44 years (n=60)** | **45–54 years (n=174)** | **55–64 years (n=203)** | **65–80 years (n=165)** | ***p* for trend** |
| --- | --- | --- | --- | --- | --- |
| Male; n (%) | 21 (35.0) | 67 (38.5) | 88 (43.6) | 74 (44.9) | 0.434 |
| Owns dwelling; n (%) | 49 (81.7) | 151 (86.8) | 180 (88.7) | 157 (95.2) | 0.013 |
| Australian/NZ; n (%) | 53 (88.3) | 151 (86.8) | 161 (79.3) | 127 (77.0) | 0.046 |
| Household Income; n (%) |  |  |  |  | <0.001 |
| Low, <$30 k | 2 (3.3) | 8 (4.6) | 27 (13.3) | 49 (29.7) |  |
| Lower middle, $30 to <$60 k | 10 (16.7) | 25 (14.7) | 44 (21.7) | 68 (41.2) |  |
| Upper middle, $60 to <$100k | 14 (23.3) | 41 (23.6) | 51 (25.1) | 21 (12.7) |  |
| High, ≥ $100k | 32 (53.3) | 96 (55.2) | 66 (32.5) | 14 (8.5) |  |
| Employment Status; n (%) |  |  |  |  | <0.001 |
| Full Time | 26 (43.3) | 99 (56.9) | 83 (40.9) | 18 (10.9) |  |
| Part Time | 17 (28.3) | 49 (28.2) | 50 (24.6) | 20 (12.1) |  |
| Retired | 5 (8.3) | 10 (5.8) | 49 (24.1) | 112 (67.9) |  |
| Other | 12 (20.0) | 16 (9.2) | 21 (10.3) | 15 (9.1) |  |
| BMI category; n (%) |  |  |  |  | 0.213 |
| Underweight/Normal; <25 | 25 (41.7) | 58 (33.3) | 68 (33.5) | 44 (26.7) |  |
| Overweight; 25 to < 30 | 24 (40.0) | 66 (37.9) | 91 (44.8) | 77 (46.7) |  |
| Obese; ≥30 | 11 (18.3) | 50 (28.7) | 44 (21.7) | 44 (26.7) |  |
| Self-rated health; n (%) |  |  |  |  | 0.581 |
| Excellent | 12 (20.0) | 22 (12.6) | 28 (13.8) | 15 (9.1) |  |
| Very good | 24 (40.0) | 74 (42.5) | 78 (38.4) | 74 (44.9) |  |
| Good | 18 (30.0) | 64 (36.8) | 83 (40.9) | 62 (37.6) |  |
| Fair/poor | 6 (10.0) | 14 (8.1) | 14 (6.9) | 14 (8.5) |  |
| Alcohol Intake; n (%) ^a^ |  |  |  |  | 0.085 |
| Low | 2 (3.3) | 15 (8.6) | 25 (12.3) | 28 (17.0) |  |
| Normal | 43 (71.7) | 121 (69.5) | 123 (60.6) | 104 (63.0) |  |
| High | 8 (13.3) | 17 (9.8) | 29 (14.3) | 21 (12.7) |  |
| Severe | 7 (11.7) | 21 (12.1) | 26 (12.8) | 12 (7.3) |  |
| Family history of diabetes; n (%) | 13 (21.7) | 48 (27.6) | 70 (34.5) | 41 (24.9) | 0.108 |
| Center for Epidemiologic Studies Depression Scale (0–20) |  |  |  |  | 0.633 |
| No symptoms (<10) | 53 (88.3) | 163 (93.7) | 183 (90.2) | 152 (92.1) |  |
| Mild symptoms (10-14) | 4 (6.7) | 5 (2.8) | 14 (6.9) | 9 (5.5) |  |
| Severe symptoms (>14) | 3 (5.0) | 6 (3.5) | 6 (3.0) | 4 (2.4) |  |
| Physical Function variables; median (25^th^, 75^th^) |  |  |  |  |  |
| Timed up and Go (s) | 5.4 (4.7 to 6.0) | 5.2 (4.6 to 5.9) | 5.6 (5.0 to 6.5) | 6.3 (5.5 to 7.30) | <0.001 |
| Knee extensor strength test (kg) | 29.1 (20.4 to 38.5) | 24.2 (16.6 to 36.0) | 25.1 (17.2 to 33.8) | 20.7 (13.7 to 29.6) | <0.001 |
| Physical function (PF-10) ^b^ | 100 (95 to 100) | 95 (85 to 100) | 90 (75 to 95) | 85 (70 to 90) | <0.001 |
| Activity variables; mean (SD) ^c^ |  |  |  |  |  |
| Sitting (all), h/day | 8.7 (1.8) | 8.7 (1.7) | 8.5 (1.9) | 9.1 (1.7) | 0.026 |
| Prolonged Sitting, h/day ^d^ | 3.9 (1.8) | 3.8 (1.5) | 3.8 (1.7) | 4.6 (1.8) | <0.001 |
| Standing, h/day | 4.8 (1.6) | 4.9 (1.5) | 5.0 (1.5) | 4.7 (1.4) | 0.168 |
| Stepping (all) h/day | 2.1 (0.7) | 2.0 (0.6) | 2.1 (0.7) | 1.9 (0.6) | 0.008 |
| Light stepping, h/day ^e^ | 1.1 (0.4) | 1.0 (0.3) | 1.1 (0.4) | 0.1 (0.4) | 0.049 |
| MVPA stepping, h/day ^f^ | 1.1 (0.4) | 1.0 (0.4) | 1.0 (0.4) | 0.1 (0.4) | 0.001 |
| Sit-stand transitions, n/day ^g^ | 55.1 (13.9) | 55.6 (14.4) | 54.3 (14.6) | 49.0 (14.9) | <0.001 |

This is the S2 Table legend.

^a^ low = 0 g/day, normal = <25 g/day (men) & < 15 g/day (women), high = 25 to <45 g/day (men) & 15 to < 25 g/day (women), severe = ≥45 g/day (men) & ≥25 g/day (women); ^b^ self-reported physical function obtained from the 10 physical function specific items in the SF-36 quality of life questionnaire; ^c^ All objective activity variables standardised for waking wear time by residuals method; ^d^ ≥30 minutes uninterrupted sitting; ^e^ Light stepping is <3 METs; ^f^ MVPA (moderate-to-vigorous physical activity) stepping is at ≥ 3 METs; ^e^ Sit-stand transitions adjusted (by residuals method) for sitting time.
