# Supplementary material for: Associations of Monitor-Assessed Activity with Performance-Based Physical Function
Source: PLoS One. 2016 Apr 13;11(4):e0153398. doi: 10.1371/journal.pone.0153398 (PMC4830578; doi:10.1371/journal.pone.0153398)
Supplement: S3 Table — (DOCX) [file pone.0153398.s003.docx]

**S3 Table. Associations of sitting, prolonged sitting, standing, stepping, light stepping, MVPA stepping and sit-stand transitions with the 8ft Timed Up and Go (TUG-8) and Knee Extensor Strength (KES) test in Australian men (n=250) and women (n=352) aged 36–80 years (AusDiab 2011/12).**

| **Activity** ^a^ | **Sex** | **TUG-8 completion time (seconds)** ^b^ | ***p*-value** | **KES linear regression (kilograms)** ^c^ | ***p*-value** |
| --- | --- | --- | --- | --- | --- |
|  |  | **RR (95% CI)** |  | **β (95% CI)** |  |
| Sitting (all) h/day | Men | 1.01 (0.99 to 1.03) | 0.991 | -0.51 (-1.61 to 0.59) | 0.627 |
|  | Women | 1.01 (1.00 to 1.02) |  | -0.18 (-0.67 to 0.31) |  |
| Prolonged Sitting, h/day ^d^ | Men | 1.00 (0.98 to 1.02) | 0.605 | -0.65 (-1.68 to 0.38) | 0.493 |
|  | Women | 1.01 (0.99 to 1.02) |  | -0.23 (-0.81 to 0.35) |  |
| Standing, h/day | Men | 1.00 (0.98 to 1.02) | 0.588 | -0.01 (-1.28 to 1.26) | 0.958 |
|  | Women | 0.99 (0.98 to 1.00) |  | 0.03 (-0.54 to 0.59) |  |
| Stepping (all), h/day | Men | 0.97 (0.91 to 1.03) | 0.439 | 3.55 (0.91 to 6.19)** | 0.152 |
|  | Women | 1.00 (0.96 to 1.03) |  | 1.37 (-0.15 to 2.89) |  |
| Light (<3 MET) stepping, h/day | Men | 0.99 (0.89 to 1.09) | 0.720 | 4.60 (-0.52 to 9.71) | 0.308 |
|  | Women | 0.97 (0.92 to 1.02) |  | 1.67 (-0.72 to 4.06) |  |
| MVPA (≥3 MET) stepping, h/day | Men | 0.93 (0.84 to 1.02) | 0.125 | 5.26 (1.27 to 9.24)* | 0.220 |
|  | Women | 1.02 (0.95 to 1.09) |  | 2.40 (-0.70 to 5.50) |  |
| Sit-stand transitions, 15/day ^e^ | Men | 1.01(1.00 to 1.03) | 0.299 | 0.88 (-0.19 to 1.96) | 0.522 |
|  | Women | 0.99 (0.97 to 1.02) |  | 0.43(-0.56 to 1.41) |  |

This is the S3 Table legend.

^a^ All objective activity variables standardised for worn waking time; ^b^ Back-transformed from log-transformed outcome as Relative Rate (RR) with 95% confidence interval (CI); adjusted for age (years), sex (male/female), self-rated health (excellent, very good, good, fair/poor), depressive symptoms (none, mild, severe) and alcohol intake (none/low, normal, high, severe); ^b^ Prolonged sitting = sitting uninterrupted in ≥30 minute bouts at a time; ^c^ Regression coefficient (β) with 95% confidence interval (CI) that adjusts for age (years), sex (male/female), self-rated health (excellent, very good, good, fair/poor), employment status (full time, part time, retired, other) and thigh length (cm) and correct for clustering/stratification (linear regression, STATA ‘survey commands’); ^d^ ≥30 minutes uninterrupted sitting; ^e^ Sit-stand transitions adjusted for sitting time; p-value for interaction by gender; * p<0.05, ** p<0.01 ***p<0.001 for association of activity with physical function within men or women
