# Supplementary material for: Associations of Monitor-Assessed Activity with Performance-Based Physical Function
Source: PLoS One. 2016 Apr 13;11(4):e0153398. doi: 10.1371/journal.pone.0153398 (PMC4830578; doi:10.1371/journal.pone.0153398)
Supplement: S4 Table — (DOCX) [file pone.0153398.s004.docx]

**S4 Table. Associations of stepping, light stepping, MVPA stepping and sit-stand transitions with the 8ft Timed Up and Go (TUG-8) and Knee Extensor test (KES) within various age-groups in Australian adults aged 36–80 years.**

|  | **Relative Rate or Regression coefficient (95% Confidence Interval)** | | | | p-value |
| --- | --- | --- | --- | --- | --- |
|  | 35-44 years (n=60) | 45–54 years (n=174) | 55–64 years (n=203) | 65–80 years (n=165) |  |
| **8ft Timed up and Go (s) ^a^** |  |  |  |  |  |
| Sitting, h/day | 1.02 (1.00 to 1.04) | 1.01 (0.99 to 1.03) | 0.99 (0.98 to 1.01) | 1.01 (0.98 to 1.04) | 0.310 |
| Prolonged sitting, h/day | 1.01 (0.98 to 1.03) | 1.02 (1 to 1.03) | 0.98 (0.96 to 1.01) | 1.01 (0.98 to 1.04) | 0.096 |
| Standing, h/day | 0.97 (0.95 to 0.99)** | 0.99 (0.97 to 1.01) | 1.00 (0.98 to 1.03) | 1.00 (0.97 to 1.04) | 0.154 |
| Stepping (all), h/day | 1.02 (0.97 to 1.07) | 0.99 (0.95 to 1.03)* | 1.02 (0.98 to 1.07) | 0.91 (0.84 to 0.98)** | 0.039 |
| Light (<3 MET) stepping, h/day | 1.02 (0.92 to 1.13) | 0.96 (0.89 to 1.03) | 1.04 (0.97 to 1.11) | 0.89 (0.8 to 1.00)* | 0.052 |
| MVPA (≥3 MET) stepping, h/day | 1.03 (0.97 to 1.10) | 1.01 (0.94 to 1.08)** | 1.03 (0.95 to 1.12) | 0.85 (0.76 to 0.95)** | 0.021 |
| Sit-stand transitions, 15/day | 1.01 (0.96 to 1.06) | 0.98 (0.95 to 1.01) | 1.02 (0.98 to 1.05) | 1.00 (0.97 to 1.04) | 0.241 |
| **Knee Extensor Test (kg) ^b^** |  |  |  |  |  |
| Sitting (all), h/day | -1.18 (-3.43 to 1.07) | -0.51 (-1.68 to 0.66) | 0.21 (-0.46 to 0.88) | -0.37 (-1.23 to 0.49) | 0.560 |
| Prolonged sitting, h/day | -1.26 (-3.28 to 0.77) | -0.87 (-2.24 to 0.5) | 0.07 (-0.91 to 1.04) | -0.23 (-0.94 to 0.48) | 0.591 |
| Standing, h/day | 1.21 (-0.86 to 3.29) | 0.07 (-1.13 to 1.27) | -0.59 (-1.28 to 0.11) | 0.16 (-0.8 to 1.12) | 0.270 |
| Stepping (all), h/day | 2.10 (-3.94 to 8.14) | 3.94 (0.95 to 6.92)* | 1.47 (-0.85 to 3.79) | 1.95 (-1.17 to 5.07) | 0.660 |
| Slow (<3 MET) stepping, h/day | 3.50 (-7.25 to 14.24) | 4.45 (-2.39 to 11.29) | 2.32 (-1.53 to 6.17) | 2.32 (-1.53 to 6.17) | 0.888 |
| Fast (≥3 MET) stepping, h/day | 2.59 (-5.82 to 10.99) | 6.27 (2.25 to 10.28)** | 2.21 (-2.22 to 6.65) | 3.74 (-1.69 to 9.16) | 0.496 |
| Sit-stand transitions, 15/day | 1.33 (-1.29 to 3.94) | 1.17 (-1.12 to 3.47) | 1.33 (-0.19 to 2.85) | -0.81 (-2.05 to 0.43) | 0.189 |

This is the S4 Table legend.

^a^ Back-transformed from log-transformed outcome as Relative Rate (RR) with 95% confidence interval (CI); adjusted for age (years), sex (male/female), self-rated health (excellent, very good, good, fair/poor), depressive symptoms (none, mild, severe) and alcohol intake (none/low, normal, high, severe); ^b^ Regression coefficient (β) with 95% confidence interval (CI) that adjusts for age (years), sex (male/female), self-rated health (excellent, very good, good, fair/poor), employment status (full time, part time, retired, other) and thigh length (cm) and correct for clustering/stratification (linear regression, STATA ‘survey commands’); p for interaction by age group; * p<0.05, ** p<0.01 ***p<0.001 for association of activity with physical function within men or women
